# The effect of an active video game intervention on physical activity, motor performance, and fatigue in children with cancer: a randomized controlled trial

**DOI:** 10.1186/s13104-019-4821-z

**Published:** 2019-11-29

**Authors:** Lotta Hamari, Liisa S. Järvelä, Päivi M. Lähteenmäki, Mikko Arola, Anna Axelin, Tero Vahlberg, Sanna Salanterä

**Affiliations:** 1 Department of Nursing Science, University of Turku, 20014 Turku, Finland; 2 Turku University Hospital, PL 52, 20521 Turku, Finland; 3 Department of Pediatric and Adolescent Medicine, Turku University Hospital, PL 52, 20521 Turku, Finland; 4 Department of Pediatrics, Tampere University Hospital, PL 2000, 33521 Tampere, Finland; 5 Department of Biostatistics, University of Turku, 20014 Turku, Finland

**Keywords:** Physical activity, Motor performance, Fatigue, Active video games, Randomized controlled trial

## Abstract

**Objective:**

To evaluate the effect of active video games in promoting physical activity and motor performance, and reducing fatigue in children with cancer. A randomized controlled trial was conducted. The intervention included playing Nintendo Wii™Fit (Nintendo Co., Ltd., Kyoto, Japan) for 30 min/day for 8 weeks. Physical activity was estimated with accelerometers, physical activity diaries and questionnaires. Movement-ABC2 and PedsQL™ were used to examine motor performance and fatigue. Intervention experiences and fidelity were examined with an interview.

**Results:**

Participants (n = 36 children with cancer, 3–16 years-old) were randomly assigned to the intervention and control groups. The median [min–max] accelerometer counts/h (500 [131–1130] vs 385 [116–1012], p = 0.63) and physical activity min/day (34 [0–150] vs 23 [0–260], p = 0.95) did not differ between the groups. Change between the pre-test and post-test regarding motor performance and fatigue was similar in both groups (motor performance p = 0.77; fatigue p = 1.00). Participants experienced playing active video games meaningful, but the intervention was not followed completely as instructed. Overall, the physical activity levels were low and one fourth of the children had or were at risk of having movement difficulties.

*Trial registration*: ClinicalTrials.gov identifier: NCT01748058 (October 15, 2012)

## Introduction

Children with cancer have to spend long periods at the hospital and are suggested to be less physically active than their healthy peers [1, 2], and lag behind in motor skill development [3]. Together with the disease and its intensive medical treatment, low levels of physical activity (PA) may lead to secondary health problems during and after cancer [4].

Increasing PA and exercise training, even during treatment, are feasible, beneficial and safe [5–8]. For better engagement, interventions should also be fun [9], flexible to allow for individual tailoring [10], and feasible both at hospital and home. The starting point of this study was the need to activate children with cancer in a fun, entertaining and effective manner. That is how we settled upon building the intervention around active video games (AVG). Playing AVGs equals to light-to-moderate PA [11].

The aim of this study was to evaluate the effect of AVGs with regard to the promotion of PA and motor performance, and reducing fatigue in children with cancer. The detailed protocol of the study is reported by Kauhanen et al. [12].

## Main text

### Methods

**Fig. 1:**
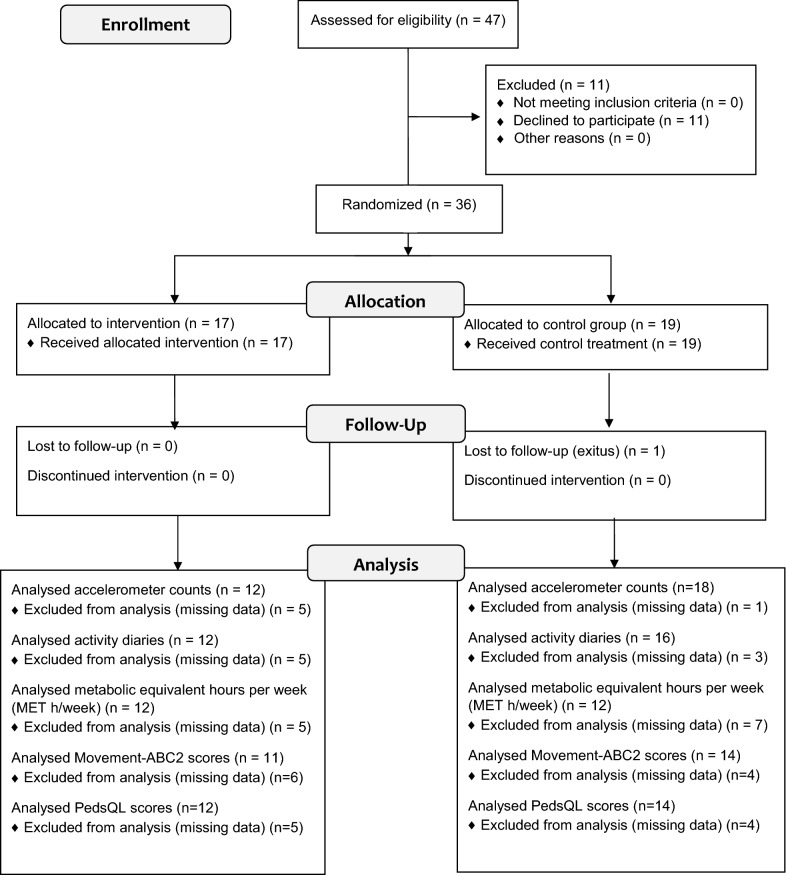
Participant flow

The study was conducted as a randomized controlled trial. Sample size was calculated based on PA measured in accelerometer counts. Mean accelerometer counts at baseline and standard deviation for both groups were set based on the study by Winter et al. [1]. The required sample size was 34 participants (80% power with a 5% significance level). The eligibility criteria for participants were (1) 3–16 years of age at the time of the diagnosis of acute lymphoblastic leukaemia or other cancer outside the central nervous system, (2) treatment included vincristine, and (3) the children were treated in either of the designated hospitals. Participant flow is reported in Fig. 1.

*Physical activity* was estimated with accelerometers, activity diaries, and two questionnaires. We used Fitbit Ultra (Fitbit Inc., San Francisco, USA) accelerometers. Fitbits were worn on the waist when being awake during the first week of the intervention. The minimum requirement for daily wear time was 8 h. The follow-up measurement was at 1 year. The results are reported as mean activity counts/h (Additional file 1).

Participants filled out *activity diary* in 10-min periods, for the first week of the intervention. The results are reported as the mean time/day spent on PAs called as “physical activity min/day”. The activity diaries were also used when evaluating the *intervention fidelity* by calculating how many minutes participants played AVGs. This is reported as “AVG playing min/week” in Table 2.

The *metabolic equivalent* (*MET*) *questionnaire* [13] assessed leisure-time PA in MET h/week pre-test and post-test. The MET questionnaire contained 3 multiple-choice questions about PA intensity, duration, and frequency.

*Motor performance* was estimated using the *Movement Assessment Battery for Children*-*2* (M-ABC2) test [14, 15] pre-test and post-test. The scores are reported in percentiles and the higher score indicates better performance.

*Fatigue* was estimated with *PedsQL™* Multidimensional Fatigue Scale questionnaire proxy reports [16]. The scores are reported on a scale from 0 to 100. The higher scores indicate lower problems.

Parents filled in the activity diary and questionnaires as a proxy reports of their child for the children below 10 years of age.

*Experiences and fidelity of the intervention* were examined with an interview. Each child was interviewed after the intervention. The children in the intervention group were asked about their experiences of the intervention and AVGs in order to know how well the intervention was followed. The interview included also a question about PA barriers in the hospital.

*Acceptability of the intervention* was evaluated based on Bowen et al. [17] and similarly than Nielsen et al. [18] by reporting the participation rate from the eligible patients and by reporting the retention rate of the participants during follow-up. We also gained information about the acceptability from the interviews.

The baseline characteristics were collected from the electronic patient records.

The intervention was managed by a physical therapist at the hospital ward and via telephone. The intervention included the use of elective Nintendo WiiFit™ games for at least 30 min/day for 8 weeks, both during hospitalization and at home. The intervention included face-to-face and written information, recommendations for PA, and the education required to use the games with age-specific instructions. In addition, the intervention group received a motivational phone call in the middle of the intervention. The control group received general written advice for PA of 30 min/day.

The first intervention meeting or the control intervention meeting were at a mean (SD) of 15.4 (13.3) days from the initial diagnosis.

The differences in the changes in scores between groups were compared with Mann–Whitney U-test. Non-parametric tests were used due to the skewed distribution of the outcome variables. The statistical computations were performed with SPSS Statistics for Windows 23 (IBM Corp., Armonk, NY). If *p*-values were less than 0.05 they were considered statistically significant. The interview data were analysed using qualitative content analysis.

### Results

The final sample size was 36/47 eligible participants (10 girls; 26 boys, participation rate 77%). The baseline characteristics of the study participants are reported in Table 1.

**Table 1:** The baseline characteristics of the study participants

	Study cohort (n = 36)	Intervention group (n = 17)	Control group (n = 19)
Age at recruitment (years)			
Mean (min–max)	7.8 (3–16)	7.8 (3–16)	7.9 (3–15)
Gender (N, female:male)	10:26	5:12	5:14
Diagnose			
Acute lymphocytic leukemia (SR:IR:HR)	17 (8:6:3)	7 (4:2:1)	10 (4:4:2)
Wilms’ tumor	2	2	0
Burkitt lymphoma	3	1	2
Non-Hodgkin lymphoma	5	3	2
Hodgkin lymphoma	3	1	2
Other neoplasm	6	3	3
Vincristine/vinblastine during first 3 months from diagnose (mg/sqm)			
Mean (STD)	10.2 (4.2)	10.2 (4.0)	10.1 (4.4)
Min–Max	1.4–17.5	3.3–16.2	1.4–17.5
Physical therapy (visits during the intervention period)			
Mean (STD)	3 (2.2)	2.4 (1.6)	3.6 (2.6)
Min–Max	0–9	1–7	0–9
Days admitted (during the intervention period)			
Mean (STD)	32.7 (13.6)	30.5 (12.1)	34.7 (14.8)
Min–Max	10–59	12–59	10–57
Hospital visits (during the intervention period)			
Mean (STD)	11.2 (7.2)	12.5 (7.4)	10.1 (7.1)
Min–Max	1–35	4–35	1–26

*SR* standard risk, *IR* intermediate risk, *HR* high risk

#### Physical activity

The difference between median (min–max) accelerometer counts for the intervention group (n = 12) 500 counts/h (131–1130) and control group (n = 18) 385 counts/h (116–1012) during the first week of the intervention was not significant (*p *= 0.63). Similarly, the difference between median of PA min/day, counted from the PA diary for the intervention group (n = 12) 34 min/day (0–150) and for the control group (n = 16) 23 min/day (0–260) was not significant (*p *= 0.95). The change in accelerometer counts between the first and the follow-up measurement did not differ between the groups (*p *= 0.22) (Table 2).

**Table 2:** Descriptive values of the outcome measures

	Intervention group		Control group		*p*-value
	N	Median (min–max)	N	Median (min–max)	
Accelerometer counts/h					
During the intervention	12	500 (131 to 130)	18	385 (116 to 1012)	0.63
At 1 year	10	524 (284 to 1381)	12	928 (462 to 1384)	0.05
Change^a^	9	212 (− 324 to1064)	12	410 (− 20 to 1087)	0.22
Active video game playing min/week	12	30 (0 to 280)	15	0 (0 to 300)	0.18
Physical activity min/day	12	34 (0 to 150)	16	23 (0 to 260)	0.95
Metabolic equivalents h/week					
At baseline	13	20 (3 to 55)	16	22 (7 to 92)	
After the intervention	8	16 (2 to 52)	12	21 (1 to 92)	
Change^a^	12	− 0.34 (− 52 to 33)	12	0 (− 34 to 15)	0.38
Movement-ABC-2 (percentile)					
At baseline	13	75 (5 to 99)	16	50 (0 to 99)	
After the intervention	14	63 (0 to 95)	17	37 (1 to 98)	
Change^a^	11	− 4 (− 47 to 54)	14	0 (− 83 to 45)	0.77
PedsQL Fatigue scores					
At baseline	14	67 (35 to 100)	17	60 (39 to 97)	
After the intervention	12	67 (40 to 92)	14	66 (47 to 90)	
Change^a^	12	4 (− 35 to 68)	14	6 (− 7 to 64)	1.00

^a^Only participants with data at both measurement points were included in the analysis

The median (min–max) MET h/week pre-test scores for the intervention group were 20 (3–55) and for the control group 22 (7–92). The median MET h/week post-test scores were 16 (2–52) for the intervention group and 21 (1–92) for the control group. The change in MET h/week did not differ between the groups (*p *= 0.38) (Table 2).

#### Motor performance

The median (min–max) M-ABC2 pre-test score in the intervention group was 75 (5–99) and post-test 63 (0–95). Median (min–max) M-ABC2 pre-test score for control group was 50 (0–99) and post-test 37 (1–98). At pre-test, 8% of the intervention group and 31% of the control group, and at post-test 21% of the intervention group and 29% of the control group had or were at risk of having movement difficulties. The change in M-ABC2 scores between the pre- and post-tests did not differ between the groups (*p *= 0.77) (Table 2).

#### Fatigue

The median (min–max) fatigue pre-test scores in the intervention group were 67 (35–100) vs 60 (39–97) in the control group. Post-test fatigue score were 67 (40–92) in the intervention group vs 66 (47–90) in the control group. The change in fatigue scores between the pre- and post-tests did not differ between the groups (*p *= 1.00) (Table 2).

#### Intervention experiences

Based on the interviews, most of the participants experienced playing AVGs to be meaningful and enjoyable. However, some guardians of the youngest children (3 years old) reported that the games were too difficult, and the child lost focus when playing. One guardian told that they did not install the games at home as there was too much going on at the beginning of the treatment. One child told getting bored to the games due to playing them so much. One child would have wanted to continue playing even after the intervention, since enjoyed it so much with friends and family. The most positive experiences about the games were reported by primary school-aged children and their guardians. The games were liked specifically when participants were instructed to stay in their own patient room and they felt not having many other possibilities to be active.

Barriers for physical activity during the hospital stays were infusion cannula (lack of ideas how to be active with it), fatigue and lack of space (too little patient rooms and lack of play/exercise area). Being stuck in the patient room or with the infusion lines, were lowering mood which in turn lessened their activity. Participants wished for gym or exercise area (including soccer goals, basketball hoops, pedal cars and tractors) on the ward. Older children told they miss organized PA for children with cancer since they could not take part to their own hobbies which they missed a lot.

#### Intervention fidelity

Based on the PA diaries 6/12 children in the intervention group and 3/15 children in the control group had played AVGs during the first week of the intervention. Only one participant in the intervention group reported that he/she had played as instructed (30 min/day = 210 min/week). The AVG minutes (median) per week per group are reported in the Table 2. Based on the PA diaries and the interviews, the intervention was not followed as recommended.

#### Acceptability of the intervention

Based on the participation rate (77%), the intervention was accepted relatively well. Even though we had missing data, no-one of the 36 participants self-wanted to withdraw from the study during the 2.5 year follow-up. Participants in the intervention group were satisfied to get the possibility to play AVGs even though they did not use them in the end as instructed.

### Discussion

In the present study, we did not find differences between the intervention and control group in PA, motor performance, or fatigue. However, the intervention was not successfully followed, and therefore definite conclusions about the intervention effects cannot be made. The intervention was well accepted based on the study participation and retention rate and mostly enjoyed based on the interviews. Adverse effects were not reported. Our results are partly in discrepancy and partly in line with the earlier findings [19, 20]. Active video gaming has been suggested to improve body coordination in survivors of childhood brain tumors [19] and to be feasible as a part of home-based exercise program in paediatric patients after hematopoietic stem cell transplantation [20].

Our intervention was likely to be too early, at least without supervision and personalized guidance, which might be one reason for poor fidelity. Nonetheless, recent evidence is reasoning early PA interventions since Nielsen et al. found 24% reduction at 3 months in physical function in children with ALL [18]. Motivating children to be physically active from the beginning of the treatment with the existing resources remains a challenge. Every-day personalized guidance to PA might not be possible in many hospitals, and therefore also independently performed daily activity is important to be promoted. Families might need psychosocial support for PA, and education regarding the benefits of PA during the cancer treatment [8].

As a clinical perspective, AVGs can be feasible addition to traditional therapies and can bring variation to exercise programs which is extremely important in engaging children in PA. Games should be further developed also in mobile devices in order to offer interventions that are attractive, up-to-date, and effective. Digital solutions are, after all, an integral part of the children’s lives today. Further research of AVGs during cancer treatment is encouraged to verify the results.

## Limitations

Limitations of the study include poor fidelity of the intervention and problems with data collection. Missing questionnaires and problems with the accelerometer data (devices got lost or the data was erroneous) resulted to data loss. At present, we also acknowledge that the data collection period with the accelerometer and activity diaries were too short to gain precise information about the main outcome and the intervention fidelity.

The common challenges with eHealth interventions, the rapid development speed of technology and relatively slow speed of implementing research, were present in our study. When the study protocol was ready and the implementation of the study began, the game console that we used was replaced with a newer one by the manufacturer. Also, those Fitbit accelerometers that we used in our data collection are no longer in production. Despite these challenges, the results and lessons learned from our study may be used in similar studies and in justifying the efforts in PA promotion in children with cancer.

## Supplementary information

**Additional file 1: Figure S1.** Individual changes for physical activity (Fitbit step counts) during the intervention and at follow-up measurement at 12 months.

## Data Availability

The datasets used and analysed during the current study are available from the corresponding author (LH) on reasonable request.

## Abbreviations

PA: physical activity
AVG: active video games
M-ABC2: Movement Assessment Battery for Children-2
MET: the metabolic equivalent
