# Supplementary material for: The effect of an active video game intervention on physical activity, motor performance, and fatigue in children with cancer: a randomized controlled trial
Source: BMC Res Notes. 2019 Nov 29;12:784. doi: 10.1186/s13104-019-4821-z (PMC6884892; doi:10.1186/s13104-019-4821-z)
Supplement: Supplementary file 1 — Additional file 1: Figure S1. Individual changes for physical activity (Fitbit step counts) during the intervention and at follow-up measurement at 12 months. [file 13104_2019_4821_MOESM1_ESM.docx]

Solid line = intervention group

Dashed line = control group

Figure S1 Individual changes for physical activity (Fitbit step counts) during the intervention and at follow-up measurement at 12 months.
